# Research on Open-Set Recognition Methods for Rolling Bearing Fault Diagnosis

**DOI:** 10.3390/s25103019

**Published:** 2025-05-10

**Authors:** Jia Xu, Yan Wang, Renyi Xu, Hailin Wang, Xinzhi Zhou

**Affiliations:** 1 School of Electronic Information, Sichuan University, Chengdu 610065, China; xmxcjjy657@163.com (J.X.); wyxst@163.com (Y.W.); 2 National Key Laboratory of Science and Technology on Reactor System Design Technology, Nuclear Power Institute of China, Chengdu 610213, China; 19508147879@163.com (R.X.); hailin_lie@163.com (H.W.)

**Keywords:** rolling bearings, Open-Set Recognition, unknown fault diagnosis, multi-scale

## Abstract

In rolling bearing fault diagnosis, when an unknown fault is present, the Closed-Set Recognition (CSR) method tends to misclassify it as a known fault. To address this issue, an Open-Set Recognition (OSR) framework is proposed for rolling bearing fault diagnosis in this study. The framework is built upon a serial multi-scale convolutional prototype learning (SMCPL) network, enhanced with an efficient channel attention (ECA) mechanism to extract the most critical fault features. The extracted features are fed into the Density Peak Clustering (DPC) module, which identifies known and unknown classes based on the computed local densities and relative distances. Finally, validation is performed on the Case Western Reserve University (CWRU) dataset, the Xi’an Jiaotong University rolling bearing accelerated life test dataset (XJTU-SY), and the Paderborn University bearing dataset (PU), Germany, and the framework is comprehensively evaluated in terms of several evaluation metrics, such as normalization accuracy and feature visualization. The experimental results show that SMCPL-ECA-DPC outperforms the comparative methods of SMCPL, CPL, ANEDL, CNN, and OpenMax and has high diagnostic performance in the identification of unknown faults.

## 1. Introduction

Rolling bearings are critical components in mechanical systems, with high demands for reliability and performance. Therefore, the timely and accurate identification and diagnosis of bearing faults are crucial [1,2]. In the past decade, data-driven fault diagnosis methods have rapidly advanced, with machine learning and deep learning being widely applied in intelligent fault diagnosis [3,4,5,6,7,8,9,10,11]. For instance, Xu et al. [5] proposed an improved multi-scale convolutional neural network model combined with a feature attention mechanism (IMS-FACNN) for diagnosing bearing faults in real wind turbines. Ruan et al. [8], based on the physical characteristics of bearing acceleration signals, guided the design of CNN hyperparameters for fault diagnosis. However, these studies [5,6,7,8] were conducted under the assumption that both the training and testing datasets come from the same distribution, resulting in effective fault diagnosis in a Closed-Set Recognition (CSR) context. In practical applications, however, due to experimental constraints, the training set may not cover all fault types and operating conditions. The studies above are prone to misclassification when encountering untrained data. Therefore, this study of Open-Set Recognition (OSR) is of great value, both for the correct classification of known fault types and for the identification of unknown fault types [12].

In traditional fault diagnosis research, CSR typically employs feature extractors, often neural networks, to obtain distinguishable features between known and unknown faults, relying on the Softmax operation for classification. Softmax uses the cross-entropy loss function (CE) for optimization, focusing on maximizing the probability distribution of known classes while neglecting the specific feature relationships between samples and the distance information between classes. This approach has inherent limitations when applied to OSR tasks.

To better solve the OSR problem, researchers have used different approaches. Bendale and Boult [13] proposed a new model layer, OpenMax, to compute the likelihood and estimate the probability that a given input belongs to an unknown class. However, this method relies on a large number of known class samples to estimate the activation vectors and distributions for each class during the training process, which places high demands on the number of known class samples, as well as computational resources. In particular, when certain category samples are few or even scarce, the performance of OpenMax may be significantly affected, as the differentiation between categories may be insufficient, resulting in less clear classification boundaries, which reduces the accuracy of the classification results. In addition, the method is more computationally intensive when the number of samples and the number of categories is large, which can significantly increase the time overhead of training and inference. Rudd et al. [14] proposed the Extreme Value Machine (EVM) based on the statistical Extreme Value Theory (EVT), which handles unknown query classes by selecting the points and distributions with the least redundancy between each category and constructing a compact probabilistic representation of the decision boundaries. However, the EVM approach has some limitations, especially when dealing with complex data distributions. EVMs usually rely on simple model assumptions, such as that the data distribution is linearly differentiable. However, in reality, data distributions are often more complex, and EVMs may lead to an oversimplification of the decision boundary, especially when faced with failure data that are highly nonlinear or multimodal. Such simplifications may not effectively capture the diversity of the data, and the performance of EVM may be degraded, especially when the data are non-uniformly distributed or have large differences between classes. EDL [15] is a learning method for self-training and inference that performs well in uncertainty quantification. However, Yu et al. [16] empirically found that it has low classification accuracy in classification tasks. In order to enhance the separation between outliers and outliers, Yu et al. proposed self-adaptive negative optimization to explicitly compress the evidential value of outliers in unlabeled data for application in open-set semi-supervised learning. However, the method relies on the high quality and quantity of labeled data and requires multiple iterations of optimization with high computational overhead.

In the diagnosis of rolling bearings, on the other hand, the fault data are often high-frequency vibration signals, characterized by different scales and frequencies, and fewer monitoring variables, usually vibration signals such as acceleration, velocity and displacement. The treatment of OSR problems in the field of rolling bearings also belongs to a type of unbalance fault diagnosis. In 2025, among the unbalanced data fault diagnosis research, Cai [17] et al. proposed the Multi-scale Dynamic Graph Mutual Information Network (MDGMIN), which includes graph generation and a multi-scale dynamic edge graph convolutional layer and mutual information learning, to solve the planetary bearing health-monitoring problem under unbalanced data by weighting and fusing specific features extracted from multi-scale dynamic edge graph convolutional layer through the attentional mechanism. Zhao [18] constructed a multi-scale Chebyshev graph convolutional layer with multi-perceptual learning as the backbone network of the multi-perceptual graph convolutional tree embedded network to capture special and shared information for the severe imbalance data of aero-engine bearings. It can be seen that for the extraction of key information from data, the capture of multi-scale information and the focusing of attention mechanisms are highly promising research tools for the present and the future.

In addition to the above methods, due to the powerful feature extraction capability and classification performance demonstrated by CNNs in the field of fault diagnosis [5,6,7,8], Yang [19] et al. proposed the Convolutional Prototype Learning (CPL) framework based on CNNs by using prototype loss (PL) as a regularization to train the data for learning. More discriminative feature representations are produced, and OSR problem solving is greatly aided by CPL’s significant improvements in intra-class compactness and inter-class separability when compared to classic CNN [20,21,22]. But it is difficult for CPL to comprehensively capture the multi-scale information and critical failure information of the samples because it relies on the convolution operation at fixed scales. To overcome the above challenges, a rolling bearing OSR framework is proposed in this paper. The framework is based on the network structure of Serial Multi-scale Convolutional Prototype Learning (SMCPL) and adds the Efficient Channel Attention (ECA) mechanism to extract the feature representations and then uses Density Peak Clustering (DPC) for recognition, which can efficiently solve the problems of insufficient multi-scale feature extraction and insufficient information interaction between channels.

The contributions of this paper are as follows:

(1) This framework is based on the serial multi-scale convolutional prototype learning network framework for feature extraction while adding an efficient channel attention mechanism so that the SMCPL-ECA network can pay more attention to the features that are important for fault identification.
(2) The Density Peak Clustering (DPC) method is used to cluster the extracted features for identification.
(3) The experiments were carried out using the Case Western Reserve University dataset (CWRU), the Xi’an Jiaotong University rolling bearing accelerated life test dataset (XJTU-SY), and the Paderborn University (PU) bearing dataset. For CWRU, 18 experiments with different known and unknown fault types were set up considering different fault diameters, and for XJTU-SY and PU, a total of 12 experiments were set up considering three different types of damage sites under the same operating conditions for testing in order to comprehensively evaluate the feasibility and superiority of the proposed framework.

The rest of this paper is organized as follows: Section 2 introduces the relevant principles of OSR, ECA, SMCPL, and DPC. Section 3 discusses the proposed framework and presents the assessment metrics. Section 4 presents the experimental data, OSR experimental setup, and model parameters. Section 5 presents the experimental results and analysis. Section 6 summarizes the conclusions of this study and future research plans.

## 2. Related Theory

### 2.1. Open-Set Recognition (OSR)

**Figure 1:**
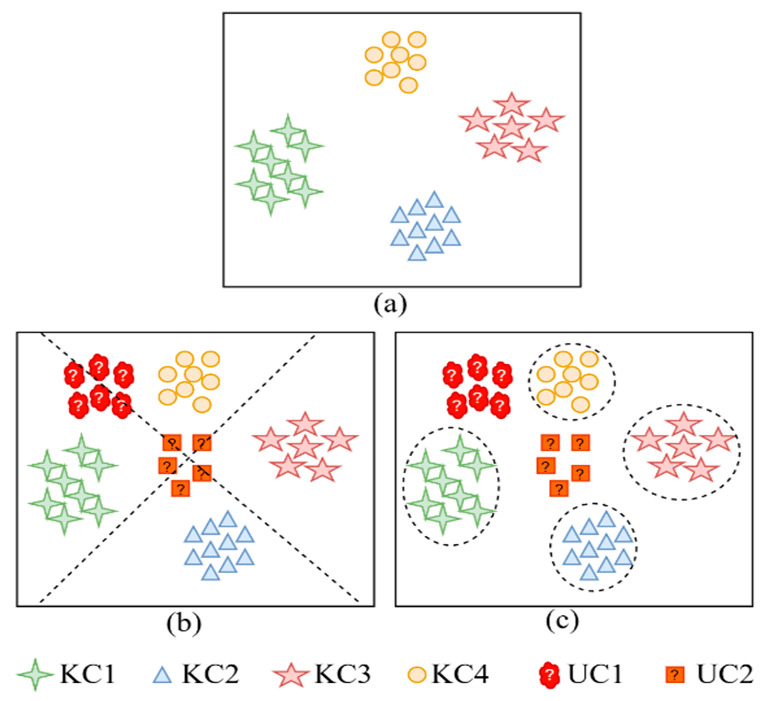
The differences between CSR and OSR: (**a**) distribution of the known class space, (**b**) CSR classification, and (**c**) OSR classification.

Research for OSR has been applied in many fields, including security monitoring [23,24], face recognition [25,26], text categorization [27,28], medical diagnosis [29], etc. However, there are still fewer experiments related to the diagnosis of industrial rolling bearing faults [20]; so, OSR based on rolling bearing faults is a worthwhile research direction. The decision boundary and the treatment of unknown samples for CSR and OSR are illustrated in Figure 1. The CSR method divides all the samples into the feature space of the known classes, and its decision boundary is unbounded, which leads to the possibility that the unknown samples may be incorrectly assigned, as shown in Figure 1b. In contrast, the OSR method defines a bounded region for each known class, and if a sample is not in the region of any known class, it is rejected as unknown.

### 2.2. Efficient Channel Attention (ECA)

This work decides to process the multi-scale features retrieved by SMCPL using the Efficient Channel Attention (ECA) mechanism [30] in order to focus more on the fault-related portion of the information among the features.

**Figure 2:**
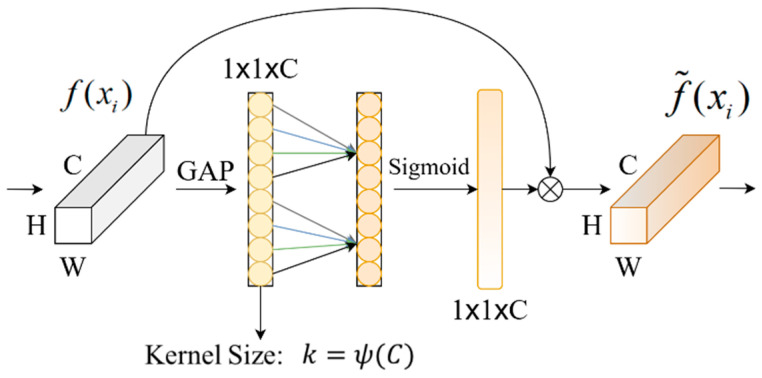
Schematic diagram of the ECA module.

Figure 2 displays the ECA module’s schematic diagram. First, global average pooling (GAP) is used to obtain each channel’s characteristics. Then, a fast 1D convolution with a convolution kernel size of k generates the channel weights and performs local channel interaction. Given the channel dimension C, the convolution kernel size k can be adaptively determined as (1), where the operation ${\left|x\right|}_{odd}$ represents the nearest odd integer to x.

(1) $$k=\psi (C)={\left|\frac{\log_{2}C}{\gamma }+\frac{b}{\gamma }\right|}_{\mathrm{odd}}$$

γ is a scaling factor that controls the weight assignment to each channel in the convolution operation. b is a bias term that is used to adjust the results of the convolution operation. In order to simplify the experimental setup, avoid adding unnecessary model complexity, and make the comparison of experimental results more concise, γ and b = 1 are set in the experiment. Finally, the sigmoid activation function is applied to generate the channel weights, which are then multiplied with the original feature map to reweight the features.

### 2.3. Serial Multi-Scale Convolutional Prototype Learning (SMCPL)

The serial multi-scale approach has lower memory and computational resource requirements compared to parallel multi-scale. Its step-by-step processing makes the model easier to control and debug, avoids the synchronization and management overheads in parallel computing, and provides more stable results when dealing with complex problems. Therefore, this paper chooses the serial multi-scale method to extract information. For an input sample $x_{i}$, the features extracted by the φ layer of Serial Multi-scale Convolutional Prototype Learning (SMCPL) are given as follows:

(2) $$f_{\varphi }(x_{i})=\omega_{\varphi }g_{\theta }(x_{i})+b_{\varphi }$$

where $\omega_{\varphi }$ and $b_{\varphi }$ denote the weights and biases of the neurons, respectively, $g_{\theta }(x)$ refers to the feature extractor that maps the raw data into the feature space. The SMCPL framework extracts multi-scale information from the data through a serial convolutional prototype learning network, which is then fused using the ECA mechanism. The final fused feature $f\left(x_{i}\right)$ is obtained by stacking along the channel dimension. This approach, in comparison to parallel network architectures, reduces computational resource consumption. The equation is as follows:

(3) $$f(x_{i})={\tilde{f}}_{a}(x_{i})+{\tilde{f}}_{b}(x_{i})+{\tilde{f}}_{c}(x_{i})$$

where 0<a<b<c≤N, with N representing the total number of layers in the network. During training, a class prototype $C_{k}$ is assigned to each known class. $C_{k}$ is derived by computing the mean of the feature vectors for each class in the training set:

(4) $$C_{k}=\frac{1}{\left|T_{k}\right|}\sum_{(x_{i},y_{i})\in T_{k}}f(x_{i})$$

where $f\left(x_{i}\right)$ denotes the feature of the sample extracted by the feature extraction network, and $\left(x_{i},y_{i}\right)$ represents the feature vector and label of the sample. $\left|T_{k}\right|$ refers to the number of samples in the training set $T_{k}$, and k indicates the number of classes. The probability that a class sample $x_{i}$ belongs to a given class in SMCPL-ECA is determined by the Euclidean distance between the sample’s mapped feature and the class prototype, as shown in (5). Here, d(·) represents the Euclidean distance, with its calculation provided in (6).

(5) $$p(y=k^{\prime }\left|x\right.)=\frac{\exp(-d(f(x_{i}),C_{k^{\prime }}))}{\sum_{k=1}^{K}\exp(-d(f(x_{i}),C_{k}))}$$

(6) $$d(f(x_{i}),C_{k^{\prime }})={\left\|f(x_{i})-C_{k^{\prime }}\right\|}_{2}^{2}$$

Based on the probability $p(y=k^{\prime }|x)$, the loss function of the network is defined as the distance cross-entropy (DCE) function, as shown below:

(7) $$l_{DCE}=-\log p(y=k)$$

**Figure 3:**
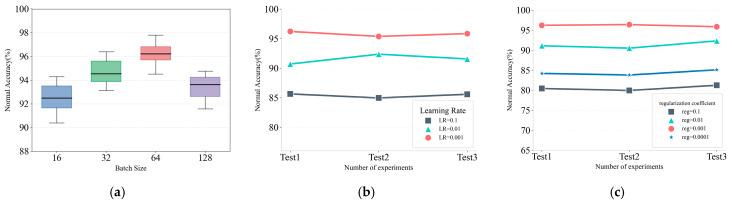
Hyperparametric sensitivity analysis. (**a**) Impact of batch size on NA. (**b**) Impact of learning rate on NA. (**c**) Impact of prototype loss weights on NA.

To address the potential overfitting issue that may arise when minimizing the DCE loss during training, SMCPL-ECA incorporates the Euclidean distance between the sample and its corresponding class prototype, referred to as the prototype loss $l_{PL}$, as a regularization term, as described in (8). The final loss function for SMCPL-ECA is defined as (9), where λ is the hyperparameter that controls the weight of the prototype loss [13]. By minimizing the objective loss function $l_{SMCPL-ECA}$, the network ultimately reduces the intra-class feature distance while maximizing the inter-class feature distance. In the experiments, λ was set to 0.001 based on the analysis of the hyperparameters in Figure 3.

(8) $$l_{PL}=\frac{1}{\left|T_{k}\right|}\sum_{i=1}^{\left|T_{k}\right|}{\left\|g_{\theta }(x_{i})-C_{k}\right\|}_{2}^{2}$$

(9) $$l_{SMCPL-ECA}=l_{DCE}+\lambda l_{PL}$$

### 2.4. Density Peak Clustering (DPC)

The Density Peak Clustering (DPC) algorithm was proposed by Rodriguez and Laio in 2014 [31]. Its core idea is to generate a decision graph with local density (ρ) on the *x*-axis and relative distance (δ) on the *y*-axis. The clustering centers are found through the decision map, and then the rest of the data are assigned to the closest clustering centers, respectively. The formulas for calculating local density ρ and relative distance δ are given in (10) and (12), respectively. For the data point $x_{i}$ with the maximum local density, the formula for its δ is provided in (13).

(10) $$\rho_{i}=\sum_{j=1,j\neq i}^{n}e^{{\left(\frac{d_{ij}}{d_{c}}\right)}^{2}}$$

(11) $$d_{ij}={\left\|x_{i}-x_{j}\right\|}^{2}$$

(12) $$\delta_{i}=\underset{j,\rho_{j}>\rho_{i}}{\min}(d_{ij})$$

(13) $$\delta_{i}=\underset{j}{\max}(d_{ij})$$

where $d_{ij}$ denotes the distance between the i-th and j-th data points, and $d_{c}$ represents the truncation distance. $d_{c}$ is used to limit the range of the neighborhood considered when calculating the local density. The value of $d_{c}$ will be adjusted according to the specific distribution of the dataset in the experiments of this paper. Based on the distribution characteristics of the distance between data points within each class, the 98% quantile of its distance value is selected as the specific parameter of $d_{c}$. The input extracted sample features that will each be aggregated after DPC clustering, and the unknown class data features are necessarily different from the known class data features; clustering will become one or more outlier points or outlier clusters as a way to distinguish between the known class and the unknown class.

## 3. Methodology

### 3.1. The OSR Fault Diagnosis Framework Based on SMCPL-ECA-DPC

**Figure 4:**
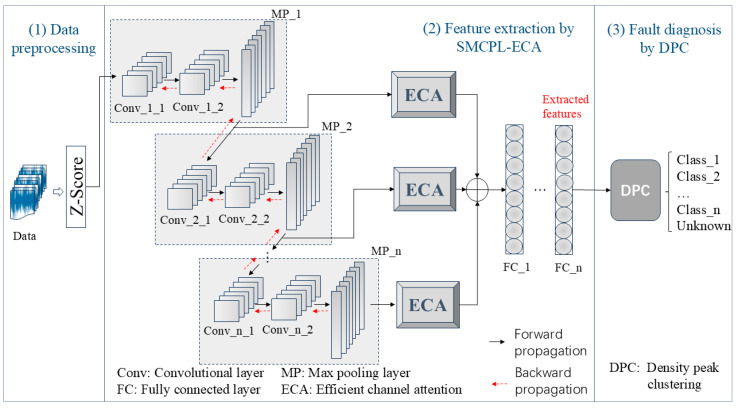
The OSR fault diagnosis framework based on SMCPL-ECA-DPC.

In this section, the proposed rolling bearing OSR fault diagnosis framework is presented. As shown in Figure 4, it consists of three modules: data preprocessing, SMCPL-ECA feature extraction, and DPC fault diagnosis.

(1) The data-preprocessing module will Z-score-normalize the raw acceleration and vibration signal data with a segment length of 1024 to eliminate the negative impact of the scale differences in the monitored variables on the model training, as shown in (14). In addition, the one-dimensional vibration signals were converted into two-dimensional images using an image conversion method [32]. The normalized 1D vectors are reconstructed into 32 × 32 2D feature maps as input.

(14) $$Z=\frac{X-\mu }{\sigma }$$

where Z represents the normalized value, X is the original data, μ denotes the mean of the original data, and σ is the standard deviation of the original data.

(2) The framework’s vital component is the SMCPL-ECA feature extraction module, which uses the data-preprocessing module’s normalized data as input and applies the multi-scale convolutional pooling operation of ECA during training to produce the known fault feature representation. For testing, mixed samples of known and unknown faults are fed into the SMCPL-ECA network architecture to obtain the extracted features of both, which are then fed into the DPC fault diagnosis module.

(3) During training, the DPC fault diagnosis module clusters the features to obtain the maximum relative distance ${max(\delta }_{i})$ of the known classes. $\lambda_{DPC}$ is a threshold value in the range of (0, 1], which is used to control the proportion of training samples misclassified by DPC. In the testing phase, DPC first calculates the relative distance $\delta_{x_{i}}$ of the test sample $x_{i}$ to the center of the clustering class of the known classes and then determines whether $\delta_{x_{i}}$ is in the range of the known classes so as to assign the sample, as shown in (15). If it falls within the known class range, the sample is classified as a known fault; otherwise, it is identified as an unknown fault. After extensive experiments to optimize and facilitate an intuitive comparison between methods, $\lambda_{DPC}$ was set to 1.

(15) $$\left\{\begin{array}{c} if\delta_{x_{i}}\leq \lambda_{DPC}\times \max(\delta_{i}),known\_class\\ if\delta_{x_{i}}>\lambda_{DPC}\times \max(\delta_{i}),unknown\_class \end{array}\right.$$

### 3.2. Evaluation Metric

In the performance evaluation of the model, both CSR and OSR performance should be considered comprehensively. This study employs three evaluation metrics: Normalized Accuracy (NA), Normalized F1 Score (NF1), and Youden’s Index (J) [12].

**Normalized Accuracy (NA)**: $A_{u}$ is defined as the proportion of correctly categorized unknown samples to the total number of unknown samples, as expressed in (16). $A_{k}$ is defined as the proportion of correctly categorized known samples to the total number of known samples, as shown in (17). NA provides a comprehensive evaluation of both Close-Set and Open-Set Recognition performance, calculated as a weighted sum of $A_{k}$ and $A_{u}$, as shown in (18).

(16) $$A_{u}=\frac{T_{u}}{T_{u}+F_{u}}$$

(17) $$A_{k}=\frac{\sum_{i=1}^{N}\left(TP_{i}+TN_{i}\right)}{\sum_{i=1}^{N}\left(TP_{i}+FP_{i}+TN_{i}+FN_{i}\right)}$$

(18) $$NA=\alpha A_{k}+(1-\alpha )A_{u}$$

In this case, **TP**, **TN**, **FP**, and **FN** stand for the true positives, true negatives, false positives, and false negatives of known samples, respectively. α is the weighting coefficient for $A_{k}$ and $A_{u}$, with a range of (0, 1). Considering that the closed-set classification ability and the unknown class detection ability in the Open-Set Recognition task are of equal importance, this paper experimentally selects α = 0.5 to balance the contribution of both in the comprehensive evaluation.

**Normalized F1 Score (NF1)**: The precision (P) and recall (R) harmonic means are called NF1. Unknown classes are treated as an extra simple class when used to assess the OSR task. It can be calculated using (21).

(19) $$P=\frac{1}{N}\sum_{i=1}^{N}\frac{TP_{i}}{TP_{i}+FP_{i}}$$

(20) $$R=\frac{1}{N}\sum_{i=1}^{N}\frac{TP_{i}}{TP_{i}+FN_{i}}$$

(21) $$NF1=2\frac{P\times R}{P+R}$$

Better categorization performance is indicated by higher **NF1** values, which range from 0 to 1. Notably, **NF1** is unaffected by changes in **TN**, although **TN** remains an important factor influencing OSR performance. To address this, Scherreik and Rigling proposed the use of the **Youden’s index (J)** for a more comprehensive evaluation of the unknown class, which is calculated from specificity (S) and recall (R) [33]. The definitions of S and J are given in (22) and (23), respectively. J denotes the ability of the algorithm to avoid failure, bounded by (−1, 1), with a higher value indicating a higher resistance to failure.

(22) $$S=\frac{TN}{FP+TN}$$

(23) J=S+R−1

## 4. Experiment

### 4.1. CWRU Experimental Data and Task Description

**Figure 5:**
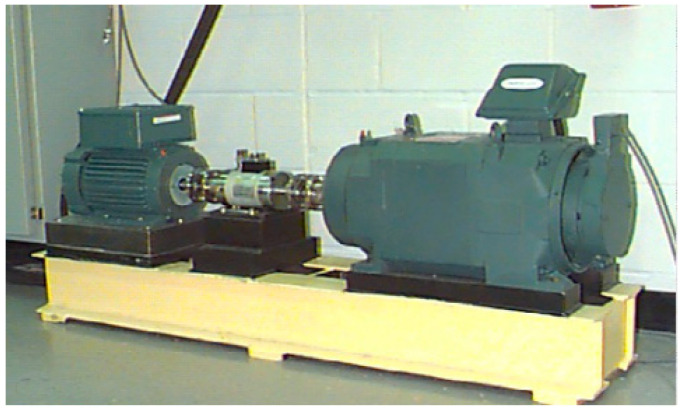
CWRU experimental equipment.

The CWRU experimental setup [34] consists of a 1.5 KW (2HP) motor, a torque transducer and decoder, and a power meter, as shown in Figure 5. Due to the extremely large amount of data in the CWRU dataset, after consideration, this paper selects the acceleration dataset with a sampling frequency of 12 kHz, a load of 1 HP, and a drive end (DE), in which three damage diameters of the inner ring failure data, the outer ring failure data, and the rolling element failure data are selected, making a total of nine types of failure data and one type of normal data. Compared with the types of failures that may occur in actual rolling bearings, the 10 types selected in this paper, although limited in number, are sufficient to verify the feasibility and effectiveness of the OSR framework based on SMCPL-ECA-DPC proposed in this paper.

Through overlapping sampling, this paper sets the number of samples for each class to 1000, with each sample presenting a length of 1024. For known classes, when partitioning the dataset, this paper divides it into a training set and a testing set in a 7/3 ratio, consisting of 700 training samples and 300 testing samples, respectively. For unknown classes, only 300 samples are selected to participate in testing alongside known class test samples, without involving them in the training phase. A detailed description of the experimental dataset is provided in Table 1.

Because motors are always in normal operation in real life, normal operating condition 9 is set as the known class when setting up the OSR tasks. In the OSR experiments with the same number of unknown classes, the faults similar to the known classes are targeted to be selected as the unknown classes on the premise that the known classes contain all fault types, and three tasks with different types of known and unknown faults are selected, and the number of unknown classes is 1~6, as shown in Table 2. For example, AE7 (with unknown classes B007(0), IR007(3), and OR007(6)) sets the class with a fault diameter of 7 mil as an unknown class, whereas AE16, AE17, and AE18 all set the class with the same fault diameter as a known class and set the classes with the other two fault diameters as unknown classes. The purpose of this setting is to deeply analyze the performance of the model under new conditions, i.e., its ability to identify unseen fault diameters, reflecting its adaptability and robustness.

### 4.2. XJTU-SY Experimental Data and Task Introduction

**Figure 6:**
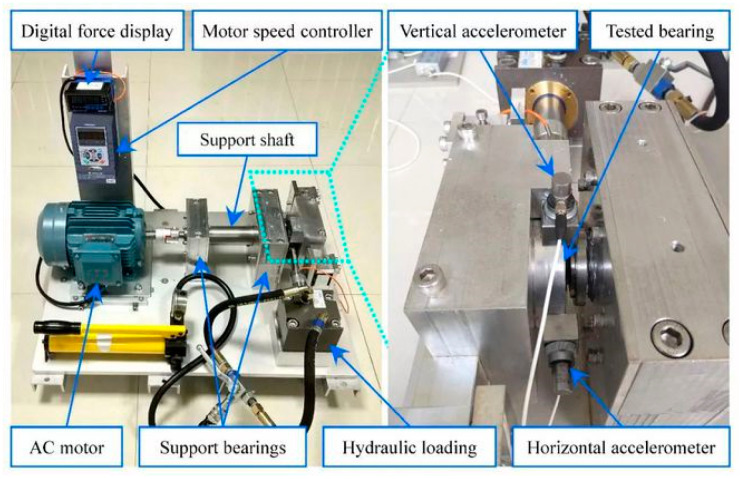
XJTU-SY experimental equipment.

The XJTU-SY dataset is a bearing failure dataset provided by Xi’an Jiaotong University, which contains data on different failure modes, such as normal operation, failure, and wear, collected in real industrial environments, and is specially used for bearing life prediction and fault diagnosis [35]. The XJTU-SY test platform consists of an AC motor, motor speed controller, rotating shaft, support bearing, hydraulic loading system, and test bearing, as shown in Figure 6. After consideration, this paper chooses the following parameters for working condition 1: a rotational speed of 2100 r/min and a radial force of 12 kN, in which three types of failure types of data are selected, which are outer ring failure data, cage failure data, and inner ring outer ring failure data at the same time.

The sample parameters, as well as those of processing, are the same as those for the CWRU data-processing flow, but the difference is that the XJTU-SY data contain two-dimensional features representing the acceleration vibration signals in the horizontal and vertical directions, respectively; so, the normalized features will be reconstructed into a 32 × 64 two-dimensional feature map as input. A detailed description of the experimental dataset is shown in Table 3. The OSR experimental setup is shown in Table 4.

### 4.3. PU Experimental Data and Task Introduction

**Figure 7:**
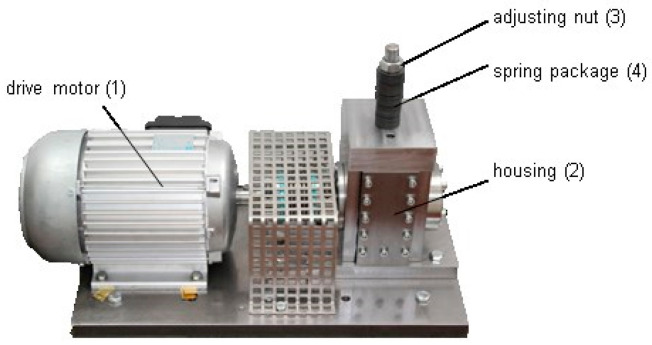
PU experimental equipment.

The PU dataset is a publicly available dataset from the University of Paderborn, Germany, constructed in 2016 to fuel research on the monitoring of bearing damage conditions in electromechanical drive systems based on motor current signals using a data-driven classification method [36]. The experimental bench shown in Figure 7 simulates a real industrial scenario, and the motor signals are susceptible to electromagnetic interference; so, the signals present a high-noise phenomenon. In this paper, the vibration signals of real damage data samples are selected for processing to verify the performance of the proposed algorithm in a complex environment. The information of the selected data samples is shown in Table 5, and the operating conditions of the bearing data samples are N09_M07_F10, i.e., the rotational speed is 900 rpm, the horsepower is 0.7 Nm, and the radial force is 1000 N. The OSR experimental settings are shown in Table 6.

### 4.4. Experimental Parameter Settings

The SMCPL-ECA-DPC model presented in this paper is implemented using the Python 3.9 programming language, with a framework built on PyTorch 2.1.0. The hyperparameter configurations are detailed in Table 7. This model selects three scale features for fusion; specifically, after obtaining three different scale features pooled by Max_pooling1, Max_pooling2, and Max_pooling3, they are input into the ECA for fusion, and the fused features are input into the Fully connected layer 1. The number of fully connected layers is set as 3. The dimension of the initialized prototype is set to the number of nodes in the output of fully connected layer 3, and this output is the data feature extracted by SMCPL-ECA, which is then input into DPC for clustering recognition. It is worth noting that since the number of samples in the training set is not the same for all tasks, the batch size will be set adaptively based on the experimental comparison of batch sizes in Figure 3. The batch size is chosen to be 64 when the number of training set samples is large and 32 when it is small. In addition, this paper employed the Adam optimizer and set the learning rate to 0.001 based on the results in Figure 3.

In this paper, CPL and SMCPL are selected for ablation experimental validation based on the proposed SMCPL-ECA-DPC model architecture, in addition to the classical CNN method, the OpenMax method, and the Adaptive Negative Evidential Deep Learning (ANEDL, 2023) [16] method for comparison experiments. The structure and parameters of the six models were optimized through extensive experiments to achieve the best performance of the models used.

## 5. Experimental Results and Analysis

This section presents the performance evaluation and analysis of the SMCPL-ECA-DPC framework, based on metrics such as NA, NF1, J, and feature visualization.

### 5.1. CWRU Experimental Results

The computational complexity of different models on the CWRU dataset is shown in Table 8. Due to the limitation of the experimental environment, this study conducts training and inference on the CPU platform; so, the average inference time for a single sample is relatively long. If GPU acceleration is used, the inference efficiency is expected to be significantly improved. Although the inference time of the SMCPL-ECA-DPC model is longer compared with simple CNN and CPL, its response time is still suitable for practical system monitoring and fault diagnosis. The sampling frequency of the CWRU dataset is 12 kHz, and each input sample contains 1024 data points, as described in this article. The sample generation time is calculated to be 1024/12,000 ≈ 85.3 ms. The inference time of the SMCPL-ECA-DPC model is 8.1 ms, which is much lower than the sample generation time of 85.3 ms. Therefore, the SMCPL-ECA-DPC model can be used for real-time monitoring of bearing health.

**Figure 8:**
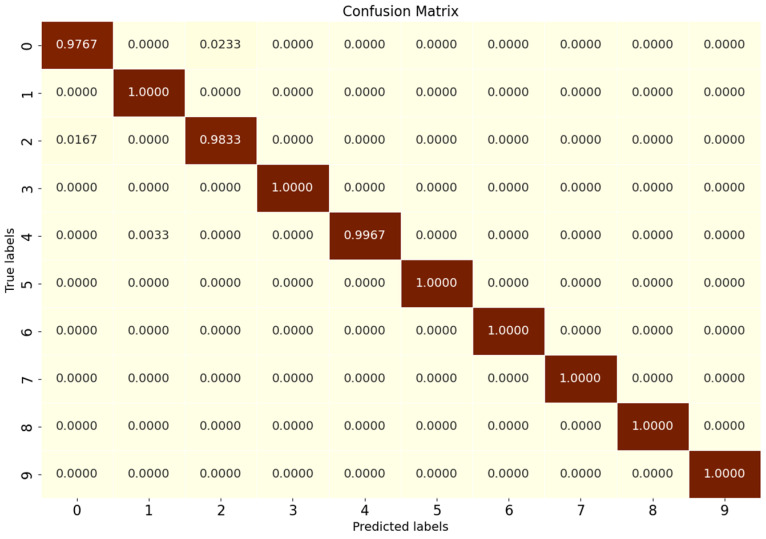
Confusion matrix for fully closed-set experiments with the SMCPL-ECA-DPC model on the CWRU dataset.

Tests were conducted according to the OSR experiment serial number in Table 2, and the results of the evaluation indexes obtained from the experiments are shown in Table 9. According to the three key performance indicators listed, the SMCPL-ECA-DPC model proposed in this paper exhibits either optimal or suboptimal performance in terms of overall performance for all 10 types of data jointly involved in the OSR experiments. Figure 8 shows the full closed-set fault diagnosis effect of SMCPL-ECA-DPC with an accuracy of 99.57% on the test set, which indicates that the model proposed in this paper is extremely effective for the extraction of critical fault signals and can provide accurate and reliable diagnosis results.

**Figure 9:**
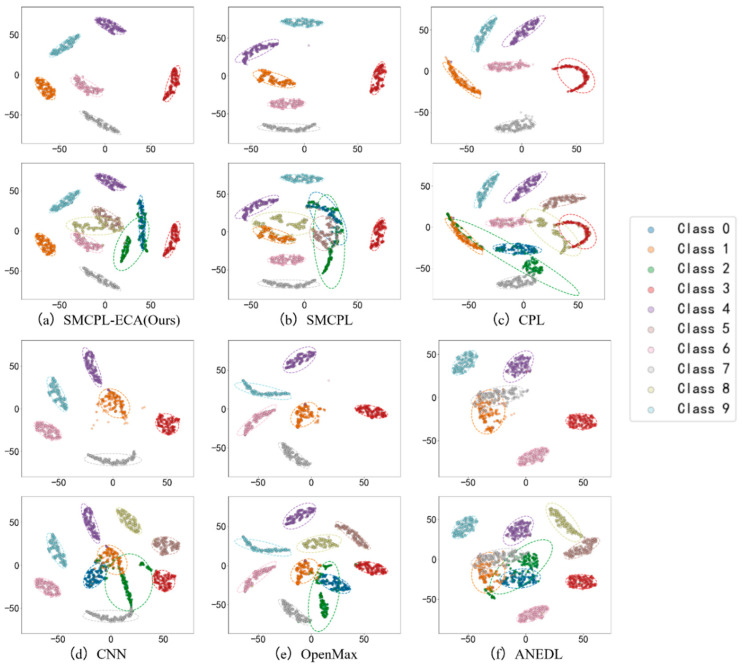
T-SNE visualization for features from AE12.

In the specific test scenario of AE2, the SMCPL-ECA-DPC model achieves a 100% recognition rate of unknown classes, which fully proves its excellent ability in feature extraction and classification tasks. For example, AE12 sets B007(0), B021(2), IR021(5), and OR021(8) as unknown classes. In this task, the proposed model is 2.71%, 7.96%, 6.81%, 13.14%, and 7.7% higher than SMCPL, CPL, ANEDL, CNN, and OpenMax on Na, respectively; on NF1, it is 0.0224, 0.068, 0.0592, 0.1048, and 0.093 higher than SMCPL, CPL, ANEDL, CNN, and OpenMax, respectively; on J, it is 0.0253, 0.0747, 0.0762, 0.1225, and 0.1031 higher than SMCPL, CPL, ANEDL, CNN, and OpenMax, respectively. In order to observe and compare the advantages and disadvantages of the six models more intuitively, the output of Fully connected layer 3 (i.e., the features extracted by the framework) is reduced from 256 dimensions to 2 dimensions for display. Figure 9 shows the spatial distribution of features extracted by AE12 using different models, and the t-distributed Stochastic Neighbor Embedding (t-SNE) algorithm is selected for the visualization algorithm [37].

**Figure 10:**
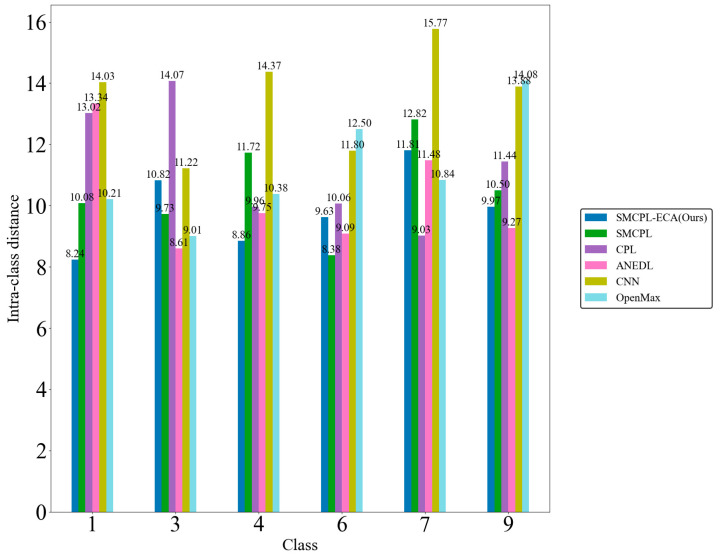
Known intra-class distances for experiment AE12.

Figure 9d shows that the CNN extracts the known fault feature B014(1) with a low degree of data dispersion and aggregation, whereas the other models for this class of data extraction still have a small number of data points outside the plotted intra-class range, but it can be clearly observed that the data points of the proposed model in this paper for the extraction of B014(1) are all centrally aggregated into a complete, continuous cluster of points with a complete distribution within the intra-class range, which is also significantly reduced according to the intra-class distance visualization in Figure 10. Intra-class distances are calculated for each class by calculating the Euclidean distance from each sample in the class to the class center and then averaging them, with the input being the Z-score-normalized features and thus dimensionless; inter-class distances are the Euclidean distances between different class centers. The number of interclass distances is extremely high, so it is not convenient to display them. Based on the intra-class distance visualization results and statistical metrics observed in Figure 10 and Table 10, SMCPL-ECA-DPC has the lowest mean (9.89), which is smaller than the comparison model’s, indicating that this model has the smallest intra-class distance and the most compact features. It is reduced by 0.64, 1.37, 0.37, 3.62, and 1.28 compared to SMCPL, CPL, ANEDL, CNN, and OpenMax, respectively. In addition to this, SMCPL-ECA-DPC has the smallest coefficient of variation (CV = 0.127), which indicates that the intra-class distances are the least fluctuating and are more robust.

**Figure 11:**
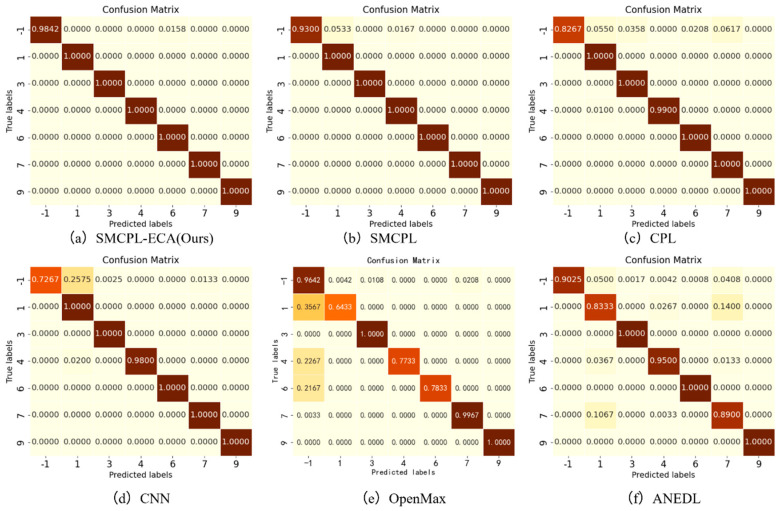
Normalized confusion matrix for experiment AE12.

After introducing the unknown class to participate in the test, by observing Figure 9d–f, it is found that among the known class and unknown class features extracted using CNN, OpenMax, and ANEDL, B007(0) and B021(2) as the unknown classes have a lower recognition rate of the unknown class because they are not involved in the training, and some of their features are distributed in the B014(1) space. For this problem, SMCPL-ECA-DPC greatly improved the separation of unknown and known classes, and the recognition performance is also substantially improved, as shown in Figure 9a. The corresponding normalized confusion matrix for AE12 is shown in Figure 11, in which the unknown class fault is labeled as −1. In the normalized confusion matrix, the rows and columns denote the true labels and predicted labels, respectively, and the numerical values in the matrix represent the correct predicted label samples’ number to the total number of true label samples. The confusion matrix classification results also confirm the superiority of the proposed model in OSR experiments.

Anomaly analysis: In some experiments, such as AE2, AE5, and AE6, the diagnostic performance of CPL is lower than that of CNN, specifically because the loss function used by CPL is different from that of CNN, which makes the same class of data closer, and because of this, misclassification may exist. Because the original data belonging to an unknown class and a known class feature distribution are too close, the training instead of the inter-class distance between the two will be so close that it cannot be separated and recognized. This is precisely why the CNN achieves optimal recognition performance in AE7, as our experimental results show that B007(0), serving as an unknown class, has feature distributions extremely similar to those of B021(2), which is also a rolling element fault, making it indistinguishable after intra-class distance reduction.

### 5.2. XJTU-SY Experimental Results

**Figure 12:**
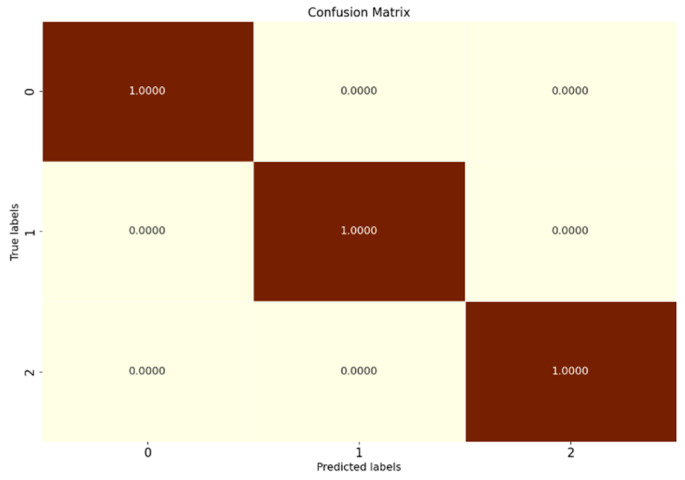
Confusion matrix for fully closed-set experiments with the SMCPL-ECA-DPC model on the XJTU-SY dataset.

Comparison tests are conducted according to the OSR experiment serial numbers in Table 4, and the results of the evaluation metrics on the XJTU-SY dataset are obtained as shown in Table 11. Figure 12 shows the full closed-set fault diagnosis results of SMCPL-ECA-DPC on this dataset with 100% test set accuracy.

**Figure 13:**
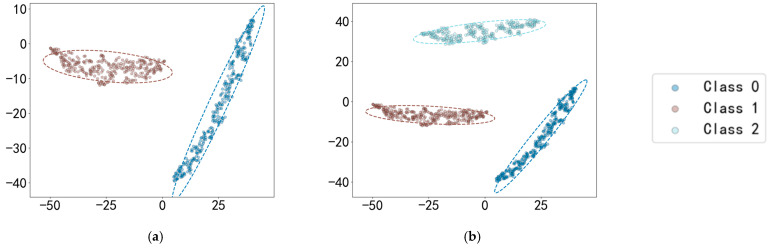
Distribution of BE3 features extracted by SMCPL-ECA-DPC. (**a**) Known classes’ distribution space for BE3. (**b**) Mixed classes’ distribution space for BE3.

**Figure 14:**
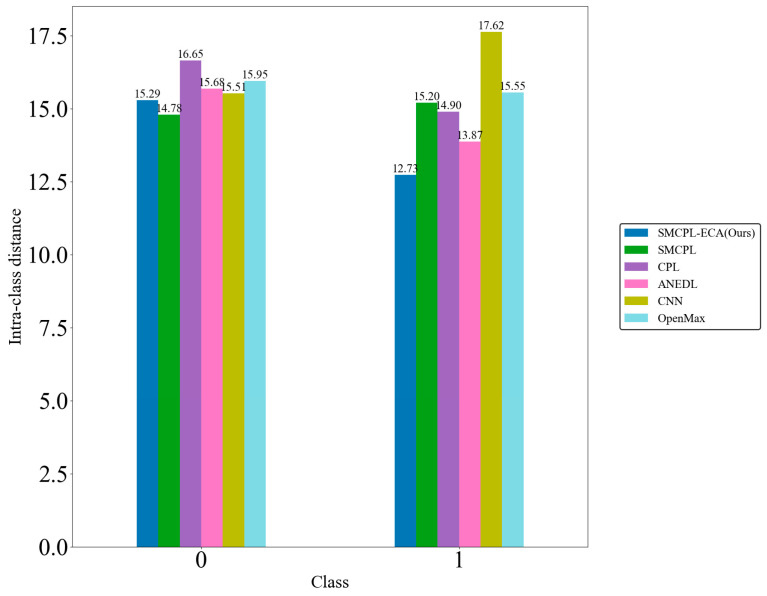
Known intra-class distances for experiment BE3.

**Figure 15:**
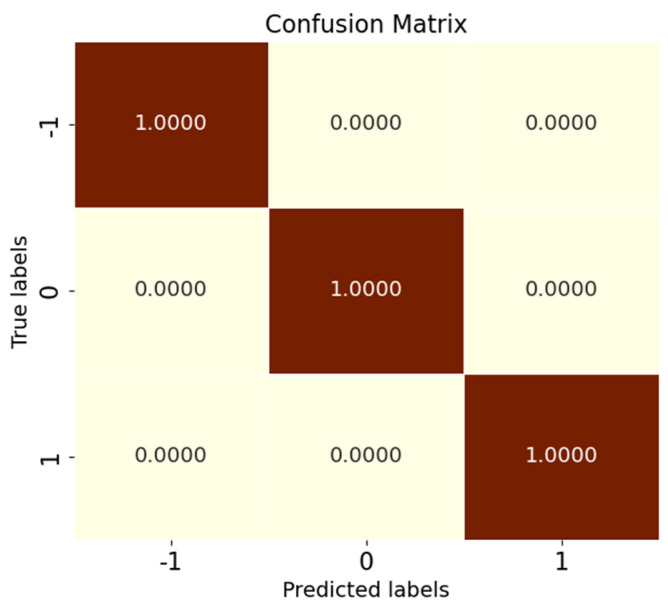
Normalized confusion matrix for SMCPL-ECA-DPC on the XJTU-SY dataset.

Table 11 demonstrates the OSR strong diagnostic performance of SMCPL-ECA-DPC on the XJTU-SY dataset, and the classification performances are all higher than those of other models. Taking BE3 (the unknown class is IORF(2)) as an example, the proposed framework in this paper achieves a 100 percent recognition rate of the unknown class with both CPL and SMCPL, and the computed NA, NF1, and J metrics are improved by 1.35%, 0.0126, and 0.0037, respectively, compared to ANEDL; by 25.67%, 0.1832, and 0.2622, respectively, compared to CNN; and by 21.42%, 0.2144, and 0.2834, respectively, compared to OpenMax. Figure 13 demonstrates the distribution of features extracted by experiment BE3 using the model of this paper. According to the intra-class distance visualization results and the statistical metrics observed in Figure 14 and Table 12, SMCPL-ECA-DPC has the lowest mean value (14.01), which is 0.98, 1.89, 0.76, 2.56, and 1.74 lower than that of SMCPL, CPL, ANEDL, CNN, and OpenMax, respectively, which indicates that the present model is better than other models for the aggregation of intra-class distances. Figure 15 represents the normalized confusion matrix of SMCPL-ECA-DPC.

Anomaly analysis: The reason for the low diagnostic performance of all methods in the BE2, BE5, and BE6 experimental results is that the distributions of ORF(0) and CF(1) in the XJTU-SY dataset are extremely similar in the feature space, which would be difficult to differentiate if both of them were not set up as known classes to be trained to increase the inter-class distance. If any one of the two classes is an unknown class, then the original part of the data distribution belonging to the unknown class during testing will be distributed in the same space with the distribution of the other class so that it cannot be separated and identified. The experiment where both are trained as known classes is BE3, analyzed as above. In experiment BE4, both ORF(0) and CF(1) are used as unknown classes, and the recognition performance of all models is very good precisely because the spatial distributions of these two classes are similar, which are very different from those of IORF(2), and thus the recognition rate of unknown classes reaches 100%.

### 5.3. PU Experimental Results

**Figure 16:**
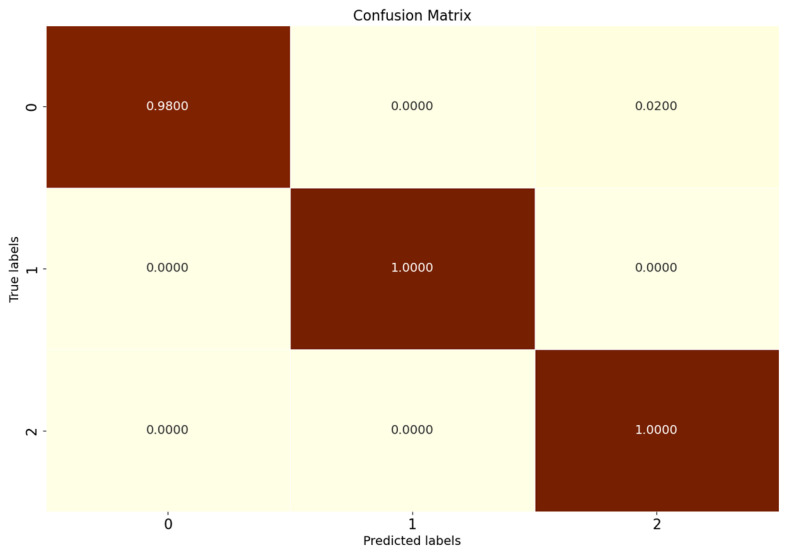
Confusion matrix for fully closed-set experiments with the SMCPL-ECA-DPC model on the PU dataset.

**Figure 17:**
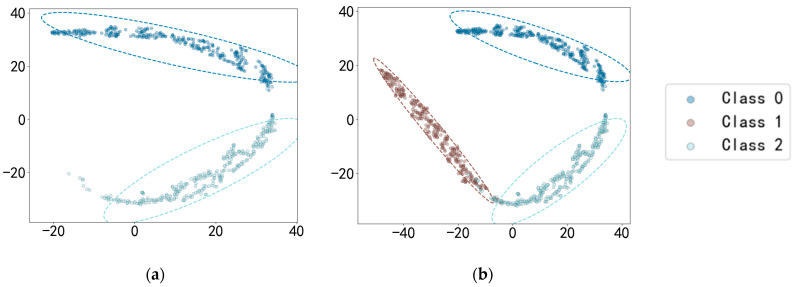
Distribution of CE2 features extracted by SMCPL-ECA-DPC. (**a**) Known classes’ distribution space for CE2. (**b**) Mixed classes’ distribution space for CE2.

**Figure 18:**
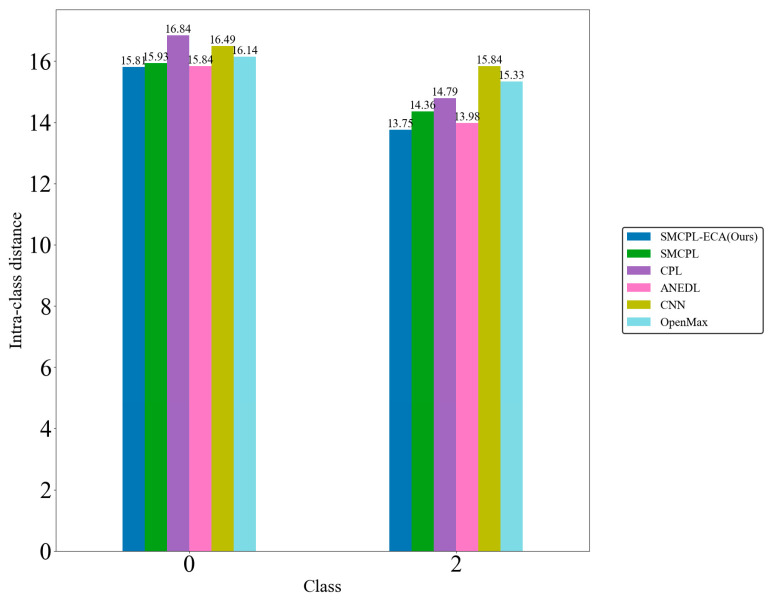
Known intra-class distances for experiment CE2.

**Figure 19:**
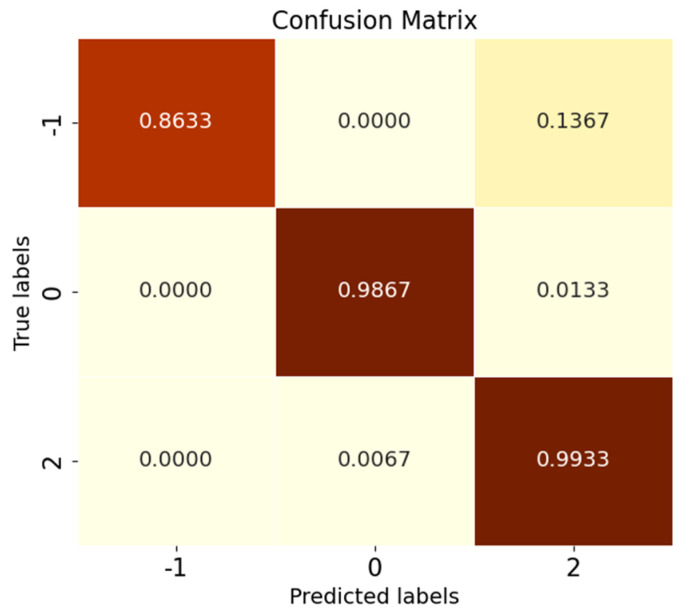
Normalized confusion matrix for SMCPL-ECA-DPC on the PU dataset.

Comparison tests are conducted according to the OSR experiment serial numbers in Table 6, and the results of the evaluation metrics on the Paderborn dataset are obtained as shown in Table 13, where the classification performance of SMCPL-ECA-DPC is higher than that of other models in all cases, which once again verifies its strong diagnostic capability for OSR on the real bearing dataset PU, which is a much larger dataset with a much higher noise level. Figure 16 shows the full closed-set fault diagnosis effect of SMCPL-ECA-DPC on this dataset, with an accuracy of 99.33% on the test set. Taking CE2 (unknown class as IR (1)) as an example, the NA, NF1, and J metrics computed by the proposed framework in this paper are higher than those of other models. Figure 17 demonstrates the feature distribution of experiment CE2 extracted using the model of this paper. According to the intra-class distance visualization results and statistical metrics observed in Figure 18 and Table 14, SMCPL-ECA-DPC has the lowest mean value (14.78), which is 0.365, 1.035, 0.13, 1.385, and 0.955 lower than that of SMCPL, CPL, ANEDL, CNN, and OpenMax, respectively, which once again verifies that the present model is more effective for intra-class distance approximation. Figure 19 represents the normalized confusion matrix of SMCPL-ECA-DPC.

Overall, according to the analysis of the experimental results of the three rolling bearing datasets, the framework based on SMCPL-ECA-DPC proposed in this paper can effectively improve the separation of known and unknown classes in the field of rolling bearings, and the diagnostic performance of the unknown classes is better than that of the comparison method, which provides support for the research of OSR.

## 6. Conclusions

To solve the problem of identifying unknown faults in the field of rolling bearings, this paper proposes an OSR fault diagnosis framework based on SMCPL-ECA-DPC. The experiments on the CWRU dataset take the classes of different fault diameters into account, and comparative experiments are also carried out on the XJTU-SY dataset and the PU dataset, and the results show that the proposed framework is higher than the other models’ in NA, NF1, and J on all the tasks, which proves that its diagnostic performance is superior to that of CPL, SMCPL, ANEDL, CNN, and OpenMax. Compared to the cross-entropy loss function (CE) used in CNN and the OpenMax layer, the use of distance cross-entropy (DCE) and prototype loss (PL) enhances the separation between known and unknown classes and outperforms the adaptive negative optimization evidence learning of ANEDL. In addition, the performance of the network on feature extraction is further strengthened by fusing multi-scale information with the introduction of an efficient channel attention mechanism, which is why this framework is able to efficiently capture the diversity and complexity of fault features and thus efficiently identify the unknown classes.

Future research will be devoted to the in-depth validation of the proposed framework on richer and more diverse rolling bearing fault datasets while continuing to optimize the structural design and hyperparameter configuration of the framework.

## Figures and Tables

**Table 1:** Description of the CWRU bearing dataset.

Fault Diameter	Health Status	Abbreviation	Number	Number of Samples
7 mil	Ball fault	B007	0	700/300
14 mil	Ball fault	B014	1	700/300
21 mil	Ball fault	B021	2	700/300
7 mil	Inner race fault	IR007	3	700/300
14 mil	Inner race fault	IR014	4	700/300
21 mil	Inner race fault	IR021	5	700/300
7 mil	Outer race fault	OR007	6	700/300
14 mil	Outer race fault	OR014	7	700/300
21 mil	Outer race fault	0R021	8	700/300
-	Normal	N	9	700/300

**Table 2:** OSR experimental setup of CWRU.

Number of Unknown Classes	Task	Known Class	Unknown Class
1	AE1	0, 1, 3, 4, 5, 6, 7, 8, 9	2
	AE2	0, 1, 2, 3, 5, 6, 7, 8, 9	4
	AE3	0, 1, 2, 3, 4, 5, 7, 8, 9	6
2	AE4	1, 3, 4, 5, 6, 7, 8, 9	0, 2
	AE5	0, 1, 2, 5, 6, 7, 8, 9	3, 4
	AE6	9, 0, 1, 2, 3, 4, 5, 6	7, 8
3	AE7	1, 2, 4, 5, 7, 8, 9	0, 3, 6
	AE8	0, 2, 3, 5, 6, 8, 9	1, 4, 7
	AE9	0, 1, 3, 4, 6, 7, 9	2, 5, 8
4	AE10	1, 4, 5, 7, 8, 9	0, 2, 3, 6
	AE11	0, 2, 3, 5, 6, 9	1, 4, 7, 8
	AE12	1, 3, 4, 6, 7, 9	0, 2, 5, 8
5	AE13	0, 3, 5, 8, 9	1, 2, 4, 6, 7
	AE14	0, 2, 4, 6, 9	1, 3, 5, 7, 8
	AE15	1, 3, 6, 7, 9	0, 2, 4, 5, 8
6	AE16	0, 3, 6, 9	1, 2, 4, 5, 7, 8
	AE17	1, 4, 7, 9	0, 2, 3, 5, 6, 8
	AE18	2, 5, 8, 9	0, 1, 3, 4, 6, 7

**Table 3:** XJTU-SY bearing dataset description.

Bearing Lifetime	Fault Element	Abbreviation	Number	Number of Samples
2 h 3 min	Outer race	ORF	0	700/300
2 h 2 min	Cage	CF	1	700/300
52 min	Inner race and outer race	IORF	2	700/300

**Table 4:** OSR experimental setup of XJTU-SY.

Number of Unknown Classes	Task	Known Class	Unknown Class
1	BE1	1, 2	0
	BE2	0, 2	1
	BE3	0, 1	2
2	BE4	2	0, 1
	BE5	1	0, 2
	BE6	0	1, 2

**Table 5:** PU bearing dataset description.

Bearing Code	Damage	Fault Element	Abbreviation	Number	Number of Samples
K001	-	Normal	N	0	700/300
KA04	fatigue:pitting	Outer race fault	OR	1	700/300
KI04	fatigue:pitting	Inner race fault	IR	2	700/300

**Table 6:** OSR experimental setup of PU.

Number of Unknown Classes	Task	Known Class	Unknown Class
1	CE1	1, 2	0
	CE2	0, 2	1
	CE3	0, 1	2
2	CE4	2	0, 1
	CE5	1	0, 2
	CE6	0	1, 2

**Table 7:** Hyperparameter settings for SMCPL-ECA-DPC.

No.	Layer Type	Kernel Number	Kernel Size	Padding	Batch Normalization	Activation	Attention	Output
0	Input	-	-	-	-	-	-	(32, 32)
1	Convolution-1-1	32	3 × 3	(2, 2)	BN	PReLU	-	(34, 34, 32)
2	Convolution-1-2	32	3 × 3	(2, 2)	BN	PReLU	-	(36, 36, 32)
3	Max-pooling-1	-	3 × 3	-	-	-	ECA	(12, 12, 32)
4	Convolution-2-1	64	3 × 3	(2, 2)	BN	PReLU	-	(14, 14, 64)
5	Convolution-2-2	64	3 × 3	(2, 2)	BN	PReLU	-	(16, 16, 64)
6	Max-pooling-2	-	3 × 3	-	-	-	ECA	(5, 5, 64)
7	Convolution-3-1	128	3 × 3	(2, 2)	BN	PReLU	-	(7, 7, 128)
8	Convolution-3-2	128	3 × 3	(2, 2)	BN	PReLU	-	(9, 9, 128)
9	Max-pooling-3	-	3 × 3	-	-	-	ECA	(3, 3, 128)
10	Fully connected layer 1	-	-	-	BN	PReLU	-	2016
11	Fully connected layer 2	-	-	-	BN	PReLU	-	1024
12	Fully connected layer 3	-	-	-	-	PReLU	-	256
13	Classifier	-	-	-	-	$l_{SMCPL-ECA}$	-	-
14	DPC	-	-	-	-	-	-	-

**Table 8:** Comparison of computational complexity of different models on CWRU dataset.

Model	FLOPs	Parameters	Average Inference Time per Sample
CPL	41.66 M	0.94 M	6.58 ms
SMCPL	43.83 M	2.62 M	8.21 ms
SMCPL-ECA-DPC(ours)	**43.83 M**	**2.62 M**	**8.1 ms**
CNN	41.66 M	0.94 M	6.05 ms
OpenMax	41.66 M	0.94 M	10.77 ms
ANEDL	44.42 M	2.74 M	12.96 ms

**Table 9:** NA, NF1, and J for all fault diameters in the CWRU dataset.

Task	Indicators	SMCPL-ECA-DPC(Ours)	SMCPL	CPL	ANEDL	CNN	OpenMax
AE1	NA(%)	**96.5**	88	80.83	92.45	80.3	69.67
	NF1	**0.98**	0.9529	0.8714	0.9432	0.8674	0.9119
	J	**0.9922**	0.9733	0.9574	0.9346	0.9563	0.9089
AE2	NA(%)	**100**	91.93	69.74	94.58	90.78	81.06
	NF1	**1**	0.9511	0.7703	0.9642	0.942	0.7675
	J	**1**	0.9778	0.9311	0.9765	0.9667	0.7041
AE3	NA(%)	**99.83**	89.69	82.39	95.43	95.02	62.76
	NF1	**0.9991**	0.9368	0.8842	0.9658	0.9692	0.781
	J	**0.9996**	0.9744	0.9569	0.9813	0.9804	0.7411
AE4	NA(%)	**95.08**	80	71.08	90.2	82.17	76.12
	NF1	**0.9685**	0.8556	0.7687	0.9247	0.8731	0.8108
	J	**0.9779**	0.91	0.8699	0.9361	0.9198	0.7829
AE5	NA(%)	**99.83**	96.63	73.04	95.84	95.08	88.35
	NF1	**0.999**	0.978	0.7914	0.9695	0.9672	0.8179
	J	**0.9992**	0.9831	0.8523	0.9762	0.9734	0.7937
AE6	NA(%)	**97.67**	83.92	51.52	89.63	73.27	73.48
	NF1	**0.9853**	0.8869	0.4918	0.9357	0.7982	0.8005
	J	**0.9895**	0.9276	0.7765	0.9583	0.8564	0.769
AE7	NA(%)	83.39	79.67	73.82	82.07	**85.96**	75.03
	NF1	0.8766	0.8408	0.7828	0.8638	**0.8949**	0.7928
	J	0.8861	0.8606	0.8198	0.8724	**0.9013**	0.7554
AE8	NA(%)	**83.5**	78.49	79.78	81.36	65.44	79.73
	NF1	**0.8775**	0.8349	0.8461	0.852	0.6995	0.7715
	J	**0.8869**	0.8514	0.8583	0.8643	0.7589	0.7478
AE9	NA(%)	**99.75**	98.28	84.67	93.46	89.25	92.03
	NF1	**0.9982**	0.9877	0.9053	0.9558	0.9191	0.9145
	J	**0.9981**	0.9882	0.8949	0.9763	0.925	0.9055
AE10	NA(%)	**95.96**	95.33	70.42	92.81	89.42	77.67
	NF1	**0.9681**	0.9630	0.7581	0.9412	0.9168	0.7929
	J	**0.9623**	0.9564	0.7239	0.917	0.9012	0.7542
AE11	NA(%)	**84.28**	77.76	64.42	80.18	69.75	72.54
	NF1	**0.8769**	0.8374	0.6954	0.8493	0.7467	0.7545
	J	**0.8530**	0.7923	0.6659	0.8369	0.7157	0.7138
AE12	NA(%)	**99.21**	96.5	91.25	92.4	86.17	91.51
	NF1	**0.9935**	0.9711	0.9255	0.9343	0.8887	0.9005
	J	**0.9926**	0.9673	0.9179	0.9164	0.8701	0.8895
AE13	NA(%)	**97.93**	96.9	91.43	96.18	90.07	88.67
	NF1	**0.9801**	0.973	0.92	0.9734	0.905	0.8813
	J	**0.9752**	0.9628	0.8972	0.968	0.8808	0.864
AE14	NA(%)	**92.9**	90.1	65.87	91.63	80.97	69.27
	NF1	**0.932**	0.9062	0.6493	0.9257	0.8321	0.6909
	J	**0.9148**	0.8812	0.5904	0.9054	0.7716	0.6312
AE15	NA(%)	**98.27**	96.6	94.73	94.56	83.03	74.5
	NF1	**0.983**	0.9679	0.9475	0.9386	0.8477	0.7593
	J	**0.9792**	0.9592	0.9368	0.9524	0.7964	0.694
AE16	NA(%)	**95.28**	93.5	88.43	92.94	85.64	84.72
	NF1	**0.9442**	0.9241	0.8873	0.9225	0.8494	0.8467
	J	**0.9292**	0.9386	0.8763	0.9042	0.8346	0.8373
AE17	NA(%)	**92.43**	91.86	87.37	90.69	84.39	86.76
	NF1	**0.9134**	0.9158	0.8673	0.9124	0.838	0.8532
	J	**0.9342**	0.9206	0.8794	0.935	0.8578	0.8649
AE18	NA(%)	**89.59**	86.39	82.85	88.16	80.41	76.57
	NF1	**0.9053**	0.858	0.8317	0.8784	0.7863	0.7804
	J	**0.9127**	0.8791	0.8268	0.897	0.792	0.7329

**Table 10:** Statistical indicators of intra-class distances for AE12.

Method	Mean (μ)	Standard Deviation (σ)	Minimum	Maximum	Coefficient of Variation (CV = σ/μ)
SMCPL-ECA-DPC	**9.89**	**1.254**	**8.24**	**11.81**	**0.127**
SMCPL	10.53	1.432	8.38	12.81	0.136
CPL	11.26	1.745	9.03	14.07	0.155
ANEDL	10.26	1.603	8.61	13.34	0.156
CNN	13.51	1.867	11.22	15.77	0.138
OpenMax	11.17	1.623	9.01	14.08	0.145

**Table 11:** NA, NF1 and J on the XJTU-SY dataset.

Task	Indicators	SMCPL-ECA-DPC(Ours)	SMCPL	CPL	ANEDL	CNN	OpenMax
BE1	NA(%)	**92.67**	91.94	91.16	90.68	90.17	91.97
	NF1	**0.9434**	0.9415	0.9376	0.9343	0.9338	0.942
	J	**0.9314**	0.9246	0.9124	0.925	0.901	0.9214
BE2	NA(%)	**75.5**	75.19	73.72	73.87	72.67	73.26
	NF1	**0.8186**	0.8083	0.7946	0.791	0.8031	0.814
	J	**0.7649**	0.7561	0.762	0.7493	0.7198	0.7274
BE3	NA(%)	**100**	**100**	**100**	98.65	74.33	78.58
	NF1	**1**	**1**	**1**	0.9874	0.8168	0.7856
	J	**1**	**1**	**1**	0.9963	0.7378	0.7166
BE4	NA(%)	**100**	**100**	**100**	**100**	**100**	99.18
	NF1	**1**	**1**	**1**	**1**	**1**	0.9465
	J	**1**	**1**	**1**	**1**	**1**	0.9837
BE5	NA(%)	**75.92**	75.53	75.18	74.88	75	72.16
	NF1	**0.6788**	0.6745	0.6694	0.6716	0.6667	0.6439
	J	**0.3578**	0.3531	0.3457	0.3484	0.3333	0.4632
BE6	NA(%)	**78.92**	77.94	77.36	77.85	76.58	71.43
	NF1	**0.7181**	0.7061	0.6973	0.714	0.6898	0.6783
	J	**0.4378**	0.4146	0.3967	0.4283	0.3756	0.4267

**Table 12:** Statistical indicators of intra-class distances for BE3.

Method	Mean (μ)	Standard Deviation (σ)	Minimum	Maximum	Coefficient of Variation (CV = σ/μ)
SMCPL-ECA-DPC	14.01	1.813	12.73	15.29	0.1294
SMCPL	14.99	0.295	14.78	15.20	0.0197
CPL	15.77	1.243	14.9	16.65	0.0778
ANEDL	14.77	1.274	13.87	15.68	0.0863
CNN	16.57	1.488	15.51	17.62	0.0898
OpenMax	15.75	0.279	15.55	15.95	0.0177

**Table 13:** NA, NF1 and J on the PU dataset.

Task	Indicators	SMCPL-ECA-DPC(Ours)	SMCPL	CPL	ANEDL	CNN	OpenMax
BE1	NA(%)	**90.83**	87.43	85.85	89.28	77.5	80.36
	NF1	**0.9285**	0.8786	0.8541	0.904	0.8089	0.8065
	J	**0.9088**	0.864	0.854	0.8956	0.775	0.792
BE2	NA(%)	**92.67**	74.75	51.25	90.46	49.33	70.41
	NF1	**0.9425**	0.7809	0.451	0.9256	0.4351	0.6769
	J	**0.9217**	0.7467	0.505	0.9183	0.4683	0.5283
BE3	NA(%)	**93.83**	90.71	88.38	91.53	82.67	83.84
	NF1	**0.9526**	0.9234	0.892	0.9375	0.8582	0.847
	J	**0.9383**	0.908	0.89	0.9213	0.8267	0.8364
BE4	NA(%)	**89.92**	88.46	84.51	87.68	78.85	73.95
	NF1	**0.86**	0.8523	0.839	0.8261	0.716	0.648
	J	**0.7311**	0.7254	0.706	0.7262	0.6372	0.4333
BE5	NA(%)	**88.33**	87.35	78.46	0.8687	60.08	64.57
	NF1	**0.8394**	0.8247	0.7316	0.8218	0.4459	0.5436
	J	**0.6889**	0.675	0.5162	0.6742	−0.0644	0.4932
BE6	NA(%)	**93.17**	91.76	84.68	92.18	63	74.3
	NF1	**0.9302**	0.9097	0.8562	0.9176	0.4937	0.7834
	J	**0.8178**	0.7954	0.726	0.8042	0.0133	0.7684

**Table 14:** Statistical indicators of intra-class distances for CE2.

Method	Mean (μ)	Standard Deviation (σ)	Minimum	Maximum	Coefficient of Variation (CV = σ/μ)
SMCPL-ECA-DPC	14.78	1.4566	13.75	15.81	0.0986
SMCPL	15.145	1.1064	14.36	15.93	0.0731
CPL	15.815	1.4459	14.79	16.84	0.0914
ANEDL	14.91	1.3174	13.98	15.84	0.0884
CNN	16.165	0.4597	15.84	16.49	0.0284
OpenMax	15.735	0.5727	15.33	16.14	0.0364

## Data Availability

The data are publicly available.
